# Obtunding Sensitive Dentine

**Published:** 1893-01

**Authors:** H. Barnes

**Affiliations:** Cleveland, O.


### Obtunding Sensitive Dentine.

BY H. BARNES, D.D.S., CLEVELAND, O.

Read before the Ohio State Dental Society, December, 1892.

*Mr. President and Gentlemen of the Ohio State Dental Society:* The subject of this paper has been chosen, not because I have any new thing to offer, but that discussion may be provoked, and if there is any new thing of value, that it may be brought to our knowledge. Who of us has not felt the need of some agent, having the power to destroy the sensitiveness of the dental organs during the time in which they are being operated upon?

In the past we have had Von Bonhorsh, Herbst, and a score of others who have heralded to the dental world a sure remedy, only to find us resorting to well-sharpened steel, after they have been tried and found wanting. We must possess a remedy which will not damage or destroy the tissue upon which we work, possessing at the same time obtunding properties.

A recent paper in *The Dental Cosmos*, on "Electricity in Dentistry," is very interesting to read, but beyond the mere statement of the writer that he believes that the time is coming when electricity will be used for obtunding sensitive dentine, there is neither reference to the subject nor help for our difficulty.

There are those who profess to have overcome the difficulty formerly existing in the use of electricity. I must confess, after repeated conversations with the patients of one of these, that there is not a little truth in their claims, but I ask the question: if this is a fact, why are they hiding so valuable a secret or discovery? Why is it not given to the profession for the benefit of humanity? Surely, if we call ours a liberal profession, the withholding of such knowledge or skill can call forth only the strongest condemnation.

We do not ask that the knowledge be given to us gratuitously, but are willing to pay, and pay well for it. We are not among those who profess the gift doctrine to the world, who, when they invent a labor-saving or pain-destroying machine, patent it, and thus contradict themselves, but, on the contrary, we hold that every man is entitled to the reward which comes from the creation of his own brain. We think him none the less a benefactor of the race, if, having discovered a means or remedy to alleviate pain, he charges a fee for such knowledge; for, gentlemen, it is what we are all doing every day of our lives, viz: charging for our knowledge.

I said, in the opening of this, that I had nothing new to advance, but I leave with you my mite, hoping it will receive at your hands a fair trial. Don't expect too much, for absolute success is not claimed. After the dam is applied, I take Dr. Black's 1-2-3-mixture, or oil of cassia, oil of wintergreen, or others of the essential oils on a pledget of cotton, placing the cotton in the cavity, and with my chip syringe, having a platinum point, draw the heated air from the lamp, and, having the nozzle of the syringe red hot, blow gently on the cotton, until the oil is driven from it.

This is done repeatedly until the cotton looks as though scorched by fire. Now, removing the cotton from the cavity, we are able to cut out quite a considerable without pain to the patient. This is especially true of the white or light brown decay found in the teeth of young children. I find this method is so successful that I employ it in all cases of this character, and seldom fail.

It is also a great help in many other cases. The little instrument made by Small's Thermal Appliance Co. of Providence, R. I., which throws a spray of heated alcohol into the cavity, is also a very good way to obtund sensitiveness of dentine, and in my judgment no office is complete without an instrument of this character.

